# Structure-function relationship of a citrus salicylate methylesterase and role of salicylic acid in citrus canker resistance

**DOI:** 10.1038/s41598-019-40552-3

**Published:** 2019-03-07

**Authors:** Caio Cesar de Lima Silva, Hugo Massayoshi Shimo, Rafael de Felício, Gustavo Fernando Mercaldi, Silvana Aparecida Rocco, Celso Eduardo Benedetti

**Affiliations:** Brazilian Biosciences National Laboratory (LNBio), Brazilian Center for Research in Energy and Materials (CNPEM), CEP, 13083-100 Campinas, SP Brazil

## Abstract

Salicylic acid (SA) and its methyl ester, methyl salicylate (MeSA) are well known inducers of local and systemic plant defense responses, respectively. MeSA is a major mediator of systemic acquired resistance (SAR) and its conversion back into SA is thought to be required for SAR. In many plant species, conversion of MeSA into SA is mediated by MeSA esterases of the SABP2 family. Here we show that the *Citrus sinensis* SABP2 homologue protein CsMES1 catalyzes the hydrolysis of MeSA into SA. Molecular modeling studies suggest that CsMES1 shares the same structure and SA-binding mode with tobacco SABP2. However, an amino acid polymorphism in the active site of CsMES1-related proteins suggested an important role in enzyme regulation. We present evidence that the side chain of this polymorphic residue directly influences enzyme activity and SA binding affinity in CsMES proteins. We also show that SA and CsMES1 transcripts preferentially accumulate during the incompatible interaction between *Xanthomonas aurantifolii* pathotype C and sweet orange plants. Moreover, we demonstrate that SA and MeSA inhibited citrus canker caused by *Xanthomonas citri*, whereas an inhibitor of CsMES1 enhanced canker formation, suggesting that CsMES1 and SA play a role in the local defense against citrus canker bacteria.

## Introduction

Salicylic acid (SA), also known as 2-hydroxybenzoic acid, is a plant hormone that has long been recognized as a defense signaling molecule involved in both local and systemic acquired resistance (SAR) against microbial pathogens in many plant species^1–4^. In response to pathogen infection, SA binds to and activates NPR1, a master regulator of the SA-mediated defense response. NPR1 functions together with the transcription factor TGA to directly promote transcription of an array of defense-related genes^4,5^.

In several plant species, SA is synthesized from chorismate by two biosynthetic routes, the isochorismate synthase (ICS) and phenylalanine ammonia-lyase (PAL) pathways. Endogenous SA can also undergo a series of chemical modifications including hydroxylation, glycosylation, methylation and amino acid conjugation^3,6–8^. Such modifications directly affect the biochemical properties of the SA derivatives, thus altering their mode of action and interaction with protein ligands. For instance, while glycosylation and amino acid conjugation are important for SA inactivation, accumulation and storage, methylation of the SA carbonyl group into a methyl ester (MeSA) greatly enhances the volatility of this molecule, making MeSA an efficient long distance and systemic defense signal^3,9,10^.

MeSA is synthesized by carbonyl methyltransferases of the SABATH family, including the S-adenosyl-L-methionine (SAM) methyl transferases (SAMT), which uses SAM as a methyl donor^11,12^. Although regarded as one of the main mediators of SAR against numerous microbial pathogens, MeSA is thought to be biologically inert and its conversion back into SA appears to be necessary for the activation of plant defenses at distal sites of pathogen attack^2,3,9,13^. This is consistent with the role played by the SA-methylesterases that catalyze the hydrolysis of MeSA into SA. These enzymes have been characterized in a few plant species, including tobacco, potato, Arabidopsis and poplar^9,14–17^.

The tobacco SABP2 protein was the first SA-methylesterase to be characterized as a low-abundance protein required for innate immunity displaying high affinity for SA^18–20^. Crystallographic studies of recombinant SABP2 bound to SA provided the structural basis for SA recognition and revealed that the active site residues S81, D210 and H238, regarded as a signature of the α/β hydrolase superfamily, act as the catalytic triad^14^. Nevertheless, the precise mechanism by which SABP2 catalyzes the cleavage of MeSA into SA remains unknown.

In previous work, we have identified a *Citrus sinensis SABP2* homologue gene (referred here as *CsMES1*) that was up-regulated in sweet orange leaves in response to infection by the citrus canker pathogens *Xanthomonas citri* (Xc) and *Xanthomonas aurantifolii* pathotype C (Xa), a Xc-related bacterium that causes canker in Mexican limes, but a hypersensitive response (HR) in sweet oranges^21,22^. Because *CsMES1* was more strongly induced during the incompatible interaction between Xa and sweet orange plants, it was postulated that it might play a role in the basal defense against the citrus canker bacteria^21^. Here, we show that the protein encoded by the *CsMES1* gene, CsMES1, displays MeSA esterase activity and that a polymorphic residue found in the active site of related CsMES1 proteins influences the MeSA esterase activity and binding affinity to SA and SA analogues. In addition, we show that SA and CsMES1 transcripts are predominantly found in sweet orange leaves in response to Xa infection and that while SA and MeSA reduced canker pustule formation caused by Xc, an inhibitor of CsMES1 promoted canker. These results suggest that CsMES1 and SA play an important role in the defense against citrus canker pathogens.

## Results

### CsMES1 is a MeSA esterase

**Figure 1:**
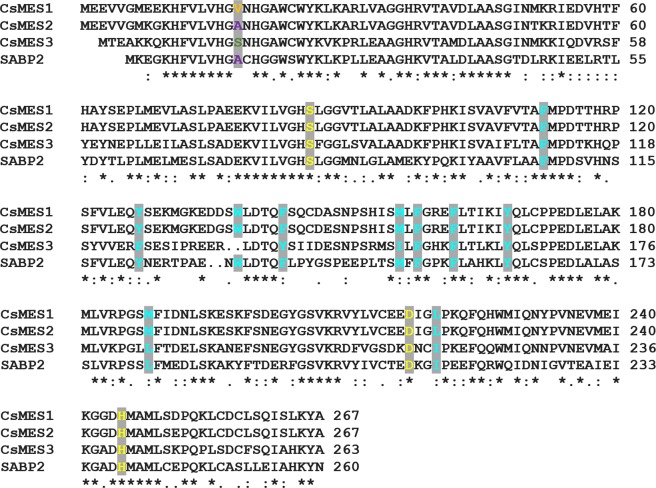
The *Citrus sinensis* CsMES variants 1–3 are related to the MeSA esterase SABP2. Protein sequence alignment showing that CsMES1 (KDO79352), CsMES2 (XP_006466663) and CsMES3 (KDO48824) are closely related to tobacco SABP2. The catalytic triad, characteristic of the α/β hydrolases and represented by residues S81, H238 and D210 in SABP2, are conserved in CsMES1, CsMES2 and CsMES3 (yellow). The hydrophobic pocket of the SABP2 active site, represented by residues in blue, is also conserved in the CsMES1-3 proteins. The polymorphic residue that belongs to the active site and which corresponds to A13 in SABP2 is indicated in orange in CsMES1 (V18), purple in CsMES2 (A18) and green in CsMES3 (S16).

The CsMES1 protein (KDO79352) was initially characterized as an ethylene-induced esterase (EIE) sharing 45 to 53% identity to hydroxynitrile lyases and polyneuridine aldehyde esterases from plants^23^. Protein sequence alignments nevertheless revealed that CsMES1 is 57% identical to and shares all the amino acid residues of the catalytic triad and hydrophobic active site with the tobacco SABP2 enzyme (Fig. 1).

**Figure 2:**
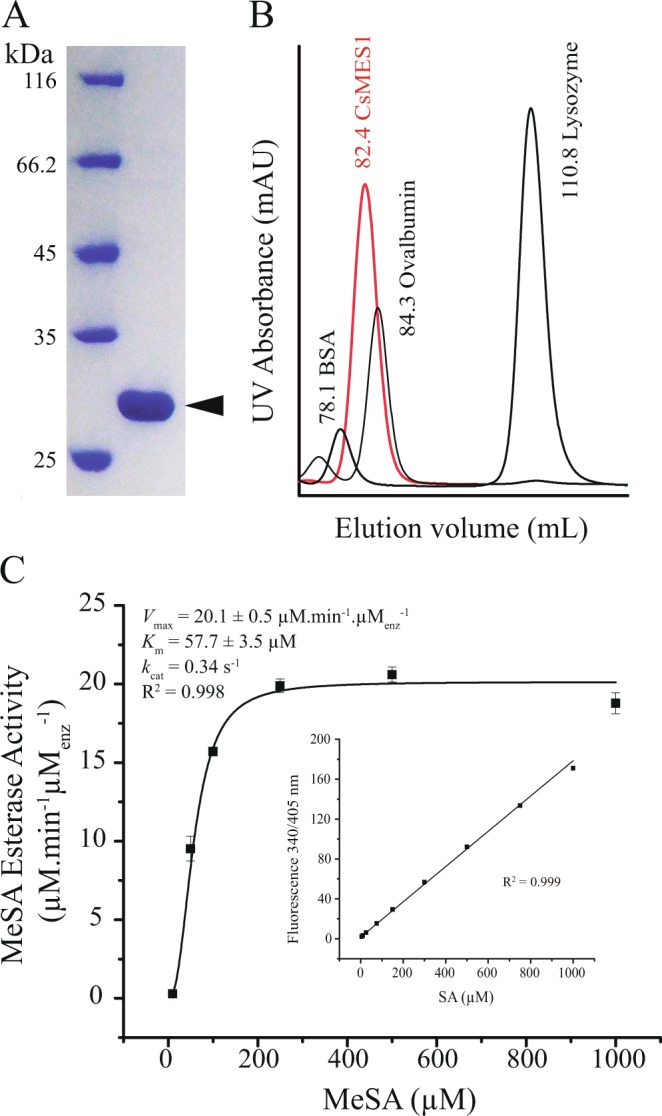
Recombinant CsMES1 is a dimer in solution and shows esterase activity upon MeSA. (**A**) SDS-PAGE showing that the recombinant CsMES1 purified by affinity and size exclusion chromatography migrates as a ~30 kDa protein. (**B**) Analytical gel filtration showing that CsMES1 elutes with an estimated molecular mass of ~54 kDa, indicating that CsMES1 is a dimer in solution. C- Purified CsMES1 shows esterase activity upon MeSA, with *k*_m_ and *V*_max_ values of ~57 and 20 µM.min^−1^.µM_enz_^−1^, respectively. The inlet graphic shows a representative calibration curve used to estimate the amount of SA converted by the enzyme as a function of MeSA concentration.

To show that CsMES1 is a MeSA esterase, the recombinant CsMES1 protein was expressed in *E. coli* cells and purified by affinity and size exclusion chromatography (Fig. 2A). CsMES1 eluted with an estimated molecular mass of 54 kDa in size exclusion chromatography, indicating that it is a dimer in solution (Fig. 2B). This protein preparation was used for the *in vitro* MeSA esterase reactions described below.

To quantify the amount of SA produced in the enzymatic reactions, a SA calibration curve was produced, based on the intrinsic SA fluorescence (Fig. 2C, inlet graphic). We found that CsMES1 shows esterase activity upon MeSA with an estimated apparent *K*_m_ and *V*_max_ of approximately 57 µM and 20 µM.min^−1^.µM_enz_^−1^, respectively (Fig. 2C). These kinetics parameters are thus comparable to those reported for other plant MeSA esterases, including SABP2^15–17^.

Next, we investigated whether CsMES1 could have any esterase activity upon methyl indoleacetic acid (MeIAA), as it is the case of SABP2 and some AtMES proteins^14,15,24^. However, we found that CsMES1 does not present any significant esterase activity upon MeIAA, even after long incubation periods (not shown).

### A polymorphic residue in the active site of CsMES proteins influences the SA binding affinity

Protein Blast searches using the CsMES1 sequence as query retrieved two additional CsMES variants (XP_006466663 and KDO48824) from the *C. sinensis* genome database, which were named CsMES2 and CsMES3, respectively. Protein sequence alignments showed that these variants also have the catalytic triad and hydrophobic active site pocket conserved, relative to SABP2 (Fig. 1).

**Figure 3:**
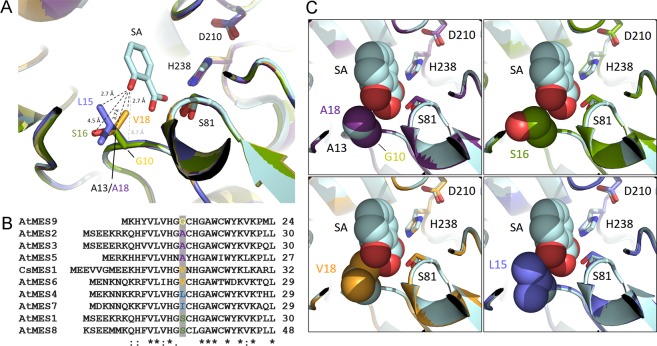
The CsMES1-3 variants show a polymorphism at the position corresponding to A13 in SABP2. (**A**) Superposition of the structural models of CsMES1 (orange), CsMES2 (purple), CsMES3 (green), AtMES4 (blue) and AtMES9 (yellow) with the crystal structure of SABP2 bound to SA (magenta). The SA molecule and catalytic triad in SABP2 (S81, D210 and H238) are indicated. The side chains of the respective amino acid residues corresponding to A13 in SABP2 are colored and their distances to the SA hydroxyl group indicated by dashed lines. (**B**) Protein sequence alignment of the N-terminal region of the AtMES family members belonging to groups I and II, showing the polymorphism at the amino acid position corresponding to V18 in CsMES1 (shown in color). (**C**) Space-filling representation of the active site in CsMES2 (purple), CsMES3 (green), CsMES1 (orange) and AtMES4 (blue) models, relative to the crystal structure of SABP2 bound to SA (magenta), showing that, in contrast to G10 (AtMES9), A18 (CsMES2) and S16 (CsMES3), the side chains of V18 in CsMES1 and L15 in AtMES4 show steric hindrance towards the SA hydroxyl group.

To structurally compare the active site of the citrus proteins with that of SABP2, we generated three-dimensional structural models of the CsMES1-3 proteins based on the crystal structure of SABP2 bound to SA^14^. A close inspection of the models revealed that the major difference among the CsMES1-3 variants is a change in the amino acid residue corresponding to A13 in SABP2, which is close to the SA hydroxyl group and is hydrogen-bonded to one of the oxygen atoms of the SA carboxyl group through its main-chain amide^14^ (Fig. 3A). The SABP2 A13 residue corresponds to V18 in CsMES1, A18 in CsMES2 and S16 in CsMES3 (Fig. 1). This polymorphism is also found among members of the AtMES family belonging to groups I and II^15,24^ where the corresponding position can be occupied by alanine, serine or valine, or alternatively by glycine, leucine or isoleucine (Fig. 3B). Because the side chains of these residues can orient towards the SA hydroxyl group (Fig. 3A), we hypothesized that a polar group at this position, such as the serine hydroxyl, might influence the SA/MeSA binding affinity. Moreover, according to our structural models, the side chains of V18 in CsMES1 and L15 in AtMES4 could also sense steric hindrance towards the SA hydroxyl group, compared to the side chains of A13/A18 in SABP2/CsMES2 (Fig. 3C). This suggested that residues with longer apolar side chains, such as V18 in CsMES1, could influence the SA/MeSA binding affinity.

**Figure 4:**
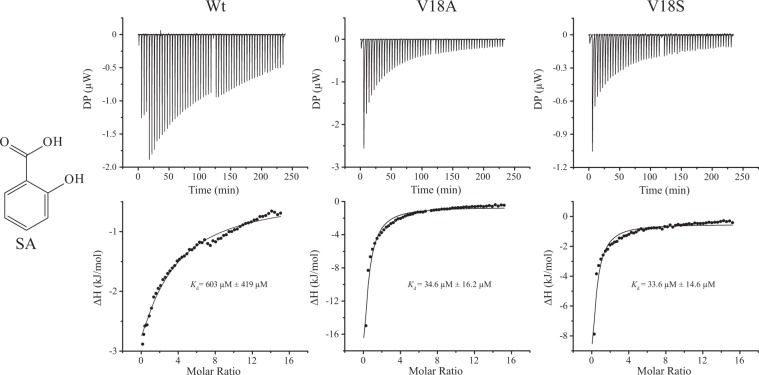
A polymorphic residue in the active site of CsMES proteins influences the SA binding affinity. ITC measurements in the presence of SA performed with the wild type CsMES1 protein (Wt) or the corresponding V18A (V18A) and V18S (V18S) mutants designed to mimic CsMES2 and CsMES3, respectively. While wild type CsMES1 shows a weak binding affinity for SA (~600 µM), the V18A and V18S mutants show binding affinities almost twenty times lower (around 30 µM), indicating that the polymorphism observed at the V18 position in CsMES1-related proteins might influence the catalytic efficiency of these enzymes.

To test this hypothesis, the V18 residue was replaced by A (V18A) or S (V18S) in CsMES1, and the recombinant proteins were analyzed by isothermal titration calorimetry (ITC) in the presence of SA, benzoic acid (BA), 3-hydroxybenzoic acid (3HBA) or 4-hydroxybenzoic acid (4HBA). We found that while the wild type CsMES1 had a low SA binding affinity of approximately 600 µM, the V18A and V18S mutant proteins showed binding affinities around 35 µM (Fig. 4), indicating that the hydrophobic side chain of valine significantly decreases the binding affinity for SA. This is consistent with the kinetics data showing that the V18A and V18S mutants have lower *V*_max_ and overall enzymatic efficiency, relative to the wild type protein, as revealed by their *K*_m_ and *k*_cat_ parameters (Table 1).

**Table 1:** Enzymatic parameters for the MeSA esterase activity displayed by wild type CsMES1 and corresponding V18A and V18S mutant proteins.

Protein	*V*_max_ (µM.min^−1^.µM_enz_^−1^)	*K*_m_ (µM)	*k*_cat_ (s^−1^)
CsMES1	22.8 ± 3.41	48.9 ± 15.63	0.38 ± 0.06
CsMES1_V18A	1.3 ± 0.02	11.7 ± 0.97	0.022 ± 3.4E-4
CsMES1_V18S	0.7 ± 0.01	16.1 ± 0.23	0.013 ± 1.6E-4

Consistent with a role in SA interaction via the ortho-hydroxyl group, we found no significant interaction or binding affinity between BA, 3HBA or 4HBA with any of the enzymes tested (Supplementary Fig. S1). Together, our results indicate that the hydroxyl group at position 2 in the SA molecule is important for substrate recognition and that the polymorphism observed at the V18 position in CsMES1-related proteins influences the catalytic efficiency of these enzymes because the side chain of these polymorphic residues directly senses the SA hydroxyl group.

### CsMES1 is inhibited by Trifluoroacetophenone

**Figure 5:**
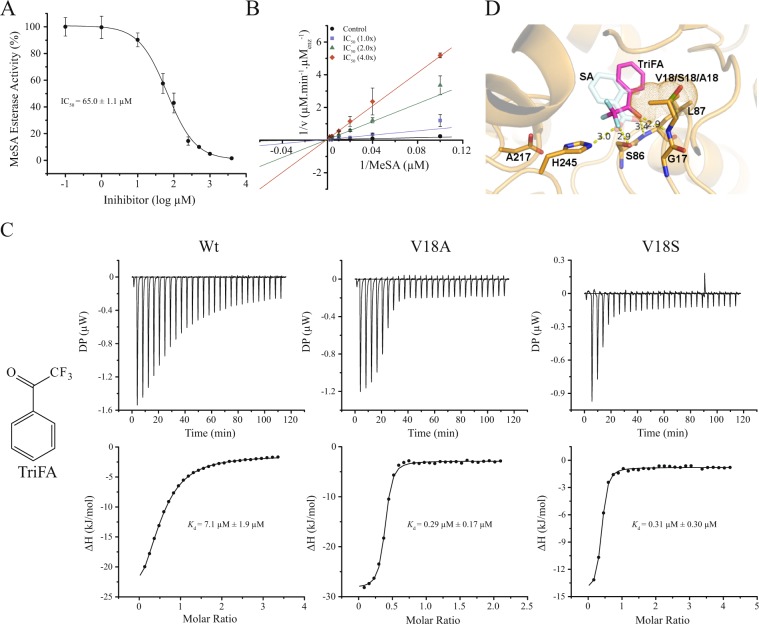
CsMES1 is inhibited by TriFA. (**A**) Dose-response curve showing that TriFA significantly inhibits the CsMES1 esterase activity (IC_50_ = 65 µM) when the substrate concentration was set to 100 µM. (**B**) Lineweaver-Burk plot showing that TriFA competes with MeSA for the CsMES1 active site in the presence of increasing amounts of TriFA (1x, 2x and 4x the IC_50_ concentration). (**C**) ITC measurements showing that TriFA binds CsMES1 with an affinity of 7.1 µM, which is approximately eighty times lower than the SA binding affinity. The biding affinity for TriFA is even lower for the CsMES1 mutant proteins V18A and V18S (~0.3 µM), suggesting that the polymorphism at this position also affects TriFA binding. (**D**) Molecular model of CsMES1 bound to TriFA (magenta) showing that TriFA mimics the binding mode of SA (light blue). In this binding conformation, the tri-Fluor atoms (cian) contact residues H245 and S86 via hydrogen bond interactions, while the carboxyl group accepts hydrogen bonds from the L87 and G17 main chain amines. The model also illustrates that the carbonyl and benzene ring of TriFA orient towards the side chain of the CsMES polymorphic residues V18/A18/S18, as it is the case of the SA hydroxyl group.

Trifluoroacetophenone (TriFA), a SA analogue, has been described as a competitive inhibitor of the tobacco SABP2 enzyme^13^. Here, we found that TriFA significantly inhibited the esterase activity of CsMES1, with an IC_50_ of ~65 µM in reactions containing 100 µM MeSA (Fig. 5A). A Lineweaver-Burk plots with increased amounts of TriFA also indicated that TriFA competes with MeSA for CsMES1 binding (Fig. 5B). Consistent with this, ITC measurements revealed that CsMES1 had a higher binding affinity for TriFA (~7.0 µM) compared to SA (Figs 5C and 4A). In addition, we found that the binding affinity of TriFA was even higher for the CsMES1 V18A and V18S mutants (~0.3 µM), compared with the wild type protein (Fig. 5C), suggesting that the V18 side chain also influences TriFA binding.

To further investigate this, we generated structural models of CsMES1, CsMES1_V18A and CsMES1_V18S mutant proteins and performed molecular docking analyses with TriFA. We found that in the three models analyzed, TriFA binds to the enzyme active site, as expected for a substrate competitor. However, TriFA assumed a slightly rotated binding conformation relative to SA, in which the tri-Fluor group contacts residues H245 and S86 via hydrogen bond interactions, while the carboxyl group accepts hydrogen bonds from the main chain amines of L87 and G17 (Fig. 5D). Such interactions are thought to stabilize the TriFA molecule in the active site pocket, which could explain the higher binding affinity observed for TriFA compared with SA. The TriFA-binding orientation found in our molecular dockings is also similar to the TriFA-binding mode proposed for the tobacco SABP2 protein^13^. However, in our docking studies, the TriFA molecule assumes a binding conformation where its carbonyl and benzene ring orient towards the side chain of the polymorphic residue just like the SA hydroxyl group (Fig. 5D). These data, therefore, support the idea that the V18 side chain experiences steric hindrance against the TriFA molecule, and this would contribute for the lower TriFA-binding affinity observed for the wild type CsMES1 protein compared to the V18A and V18S mutants.

### *CsMES1* transcripts and SA accumulate at higher levels in Xa-infected leaves

**Figure 6:**
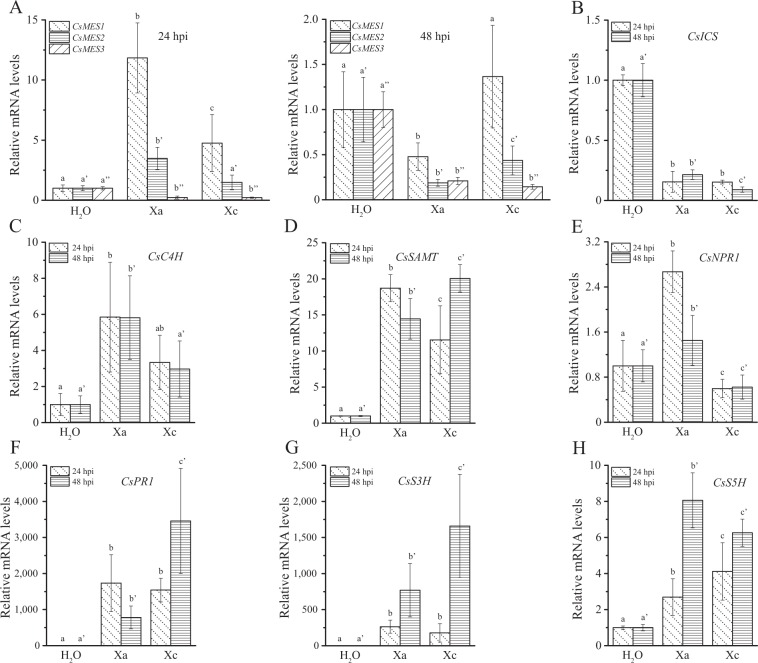
*CsMES1* transcripts accumulate during the incompatible interaction. (**A**) RT-qPCR showing the relative expression levels of *CsMES1*, *CsMES2* and *CsMES3* in sweet orange leaves infiltrated with Xa or Xc, relative to water (H_2_O), at 24 and 48 h post-inoculation (hpi). *CsMES1* is induced at higher levels by Xa, relative to Xc infection, at 24 hpi. *CsMES2* is also up-regulated by Xa at 24 hpi, but less induced than *CsMES1*. On the other hand, *CsMES1* and *CsMES2* were down-regulated by Xa and Xc at 48 hpi, whereas *CsMES3* was down-regulated by Xa and Xc both at 24 and 48 hpi. (**B**–**H**) RT-qPCR showing the relative expression levels of *CsICS*, *CsC4H*, *CsSAMT*, *CsNPR1*, *CsPR1*, *CsS3H* and *CsS5H* at 24 and 48 hpi, respectively. *CsICS* is repressed by both bacteria, whereas *CsC4H* is induced at higher levels by Xa at 24 and 48 hpi, in comparison to Xc (**B**,**C**) respectively). *CsSAMT* and *CsNPR1* are also preferentially up-regulated by Xa at 24 hpi, whereas *CsPR1*, *CsS3H* and *CsS5H* are up-regulated by both Xc and Xa (**D**–**H**) respectively). Values are the means of three independent biological replicates, each composed of three technical replicates; the error bars represent standard deviations, whereas different letters above the bars denote statistically significant differences at the 0.05 level.

In a previous gene expression study, we found that *CsMES1* transcripts were up-regulated by Xc, but more strongly induced in the incompatible interaction between Xa and sweet orange plants^21^. To confirm these observations, the relative expression levels of *CsMES1*, *CsMES2* and *CsMES3* were measured in sweet orange leaves infiltrated with Xa, Xc or water as control. In line with the microarray data^21^, *CsMES1* transcripts accumulated at higher levels in response to Xa infection 24 h post bacterial inoculation (hpi). *CsMES2* was also up-regulated by Xa at 24 hpi, but less induced than *CsMES1* (Fig. 6A). On the other hand, *CsMES1* and *CsMES2* were down-regulated by Xa and Xc at 48 hpi, whereas *CsMES3* was down-regulated by Xa and Xc both at 24 and 48 hpi (Fig. 6A). These results suggested that CsMES1, and possibly SA, could play a role in the early local defense response against citrus canker bacteria.

To investigate this further, we monitored the expression levels of the citrus genes encoding Isochorismate synthase (CsICS) and Cinnamate-4-hydroxylase (CsC4H), which are involved in SA synthesis^6,25^, S-adenosyl-L-methionine:salicylic acid carboxyl methyltransferase (CsSAMT), which converts SA into MeSA^26^, and CsS3H (SA 3-hydroxylase) and CsS5H (SA 5-hydroxylase), involved in SA homeostasis^27,28^. We also analyzed the expression levels of the SA receptor NPR1 and Pathogenesis-related protein 1 (CsPR1), a marker gene for Xc and Xa infection and SA-mediated disease resistance^5,21^. We found that although CsICS was down-regulated by Xc and Xa (Fig. 6B), suggesting that SA synthesis via the ICS pathway is repressed during infection by these bacteria, CsC4H was significantly induced by Xa at 24 and 48 hpi (Fig. 6C). We also found that CsSAMT and CsNPR1 were preferentially up-regulated by Xa at 24 hpi, whereas CsPR1, CsS3H and CsS5H were up-regulated by both Xc and Xa (Fig. 6D–H). These results indicate that although both bacteria induce changes in the SA metabolism in the early stages of infection, Xa appears to more effectively activate an SA-mediated defense response in sweet orange. Consistent with this idea, we identified SA by LC-MS/MS in three independent samples of sweet orange leaves infected with Xa or Xc at 24 and 48 hpi, but not in water-infected leaves (Supplementary Fig. S2). Moreover, judging by the area of the mass peaks, SA was more abundantly detected in Xa relative to Xc-infected leaves at 48 hpi (Supplementary Fig. S2). Conversely and consistent with the *CsS3H* expression levels, 2,3-dihydroxybenzoic acid (2,3DHBA) was found at higher levels in Xc-infected leaves at 48 hpi (Supplementary Fig. S3), indicating that Xc promotes the conversion of SA into 2,3DHBA.

### SA and MeSA reduced canker formation

**Figure 7:**
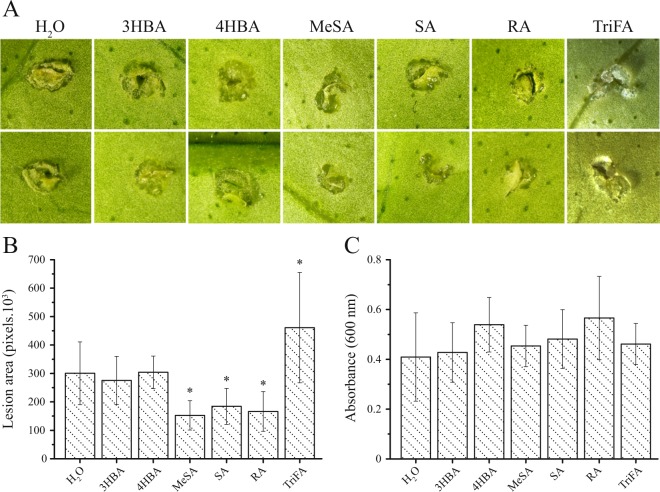
Effect of SA, MeSA, 3HBA, 4HBA, RA and TriFA on canker development. (**A**) Sweet orange leaves inoculated with Xc or water as control (H_2_O) by the pinprick method, showing that MeSA, SA and RA significantly inhibited canker pustule formation, whereas TriFA promoted cell hypertrophy and hyperplasia induced by Xc. (**B**) Relative lesion area measured by ImageJ, showing the effect of the compounds tested on canker lesion size depicted in panel A. Values are the means of forty-eight measurements performed on three independent leaves, the error bars represent standard deviations, whereas asterisks denote statistically significant differences at the 0.05 level. (**C**) Growth of Xc in culture medium supplemented with the test compounds used in this experiment showing that none of the compounds significantly affected Xc growth.

From the data presented above, we decided to investigate whether SA and SA analogues could protect sweet orange leaves from Xc infection. We also tested Rosmarinic acid (RA), which, according to our LC-MS/MS analysis, was detected at higher levels in leaves infiltrated with Xa, relative to leaves infiltrated with water or Xc (Supplementary Fig. S4). We found that while SA, MeSA and RA inhibited canker formation, TriFA promoted cell hypertrophy, whereas 3HBA and 4HBA showed no effect on pustule development (Fig. 7A,B). Because these compounds did not affect bacterial growth in culture medium (Fig. 7C), the results indicate that SA contributes to canker resistance and that CsMES1 is involved in this process, since MeSA and TriFA showed opposed effects on pustule formation.

## Discussion

*CsMES1* was firstly identified as an ethylene and wounding-induced esterase gene^23^. Subsequently, *CsMES1* was found to be preferentially up-regulated in sweet orange plants in response to Xa, relative to Xc infection^21^. Here we confirmed that *CsMES1* is mainly induced during the incompatible interaction between Xa and sweet orange, and like tobacco SABP2, is a MeSA esterase.

In addition to *CsMES1*, two highly related *CsMES* genes, *CsMES2* and *CsMES3*, are found in the *C. sinensis* genome. *CsMES2* also showed preferential induction in response to Xa infection, whereas *CsMES3* was down-regulated by Xa and Xc early after bacterial inoculation. These results suggest that the proteins encoded by the *CsMES* genes might play distinct or overlapping roles in the defense against these bacterial pathogens. In line with this notion, we noticed that CsMES1, CsMES2 and CsMES3, despite having the same catalytic triad and hydrophobic active site pocket as SABP2, show a polymorphism in the amino acid residue corresponding to A13 in SABP2, which is near the SA hydroxyl group and is hydrogen-bonded to the SA carboxyl group^14^. This polymorphism is also found among the AtMES family members where, in addition to valine (CsMES1/AtMES6), alanine (CsMES2/AtMES2-3-5), and serine (CsMES3/AtMES1-8), glycine (AtMES9), leucine (AtMES4) and isoleucine (AtMES7) are found at this position (Fig. 3B)^15,24^. Here, we provide evidence that the side chain of such polymorphic residues influences the SA binding affinity. Our data suggest that residues with shorter side chains, including alanine and serine, increase the binding affinity to SA, whereas residues with longer and apolar side chains, like valine, decrease the SA binding affinity for the enzyme. This is in line with the observation that SA strongly inhibits SABP2 and that a change from alanine to leucine at position 13 significantly reduced the feedback inhibition caused by SA without altering the MeSA esterase activity of SABP2^9,14^. Moreover, the data available for substrate specificities of AtMES proteins indicate that polymorphic residues with shorter side chains, such as in AtMES1 (S16), AtMES2 (A16), AtMES3 (A16), and AtMES9 (G10) also present significant esterase activity towards MeJA, MeIAA and p-nitrophenyl acetate, possibly because these amino acids with short side chains impose less steric hindrance on the substrate molecules^15,24^. Therefore, the polymorphism found in the MES protein family studied here seems to play an essential role not only in substrate specificity but also in the feedback inhibition caused by reaction products or substrate analogues.

These findings also show that the functional diversity of MES enzymes associated with the polymorphic SA-binding residue is more complex than previously anticipated^9^. Moreover, considering that such enzymes can display different expression patterns in response to pathogen attack, as it is the case of CsMES1, 2 and 3 in response to Xc and Xa infection, it is also possible that MES proteins could play distinct and/or complementary roles both in the local and systemic defense responses.

Like CsMES1 and 2, AtMES1, 7 and 9 were transcriptionally up-regulated by a bacterial pathogen within 24 to 72 h after bacterial inoculation^15^. However, silencing of AtMES genes in Arabidopsis compromised only SAR and not local resistance against *Pseudomonas syringae* pv. *tomato*^15^. Our data showing that TriFA inhibits CsMES1 activity and promotes canker development, and that exogenously applied SA and MeSA reduce canker formation, suggest that CsMES1 and SA play a role in the local defense response against citrus canker bacteria. This idea is supported by gene expression and metabolome analyses that show that genes directly associated with SA synthesis and signaling, including *CsC4H*, *CsSAMT* and *CsNPR1*, are significantly up-regulated by Xa at early stages of infection, and that SA also accumulates at higher levels in Xa-inoculated leaves within 48 h of bacterial inoculation. These results are thus in agreement with those of Roeschlin *et al*.^29^, who showed that a Xc variant that triggers a host defense response in *Citrus limon* induces PAL expression with concomitant SA accumulation in infected leaves. Moreover, exogenously applied SA at 0.25 mM significantly reduced canker pustule formation in Newhall navel orange^30^. Furthermore, overexpression of Arabidopsis *NPR1* increased resistance to citrus canker in different citrus cultivars^31,32^. Therefore, the data showing that SA plays an important role in the basal defense against citrus canker bacteria are also in agreement with the fact that the SA signaling was critical for the non-host defense response in Arabidopsis plants challenged with Xc^33^.

The role of SA in SAR against citrus canker bacteria has also been evidenced by studies that employed the MeSA analogue acibenzolar-S-methyl (ASM) to restrict Xc growth in greenhouse and field conditions. However, ASM was only effective to control citrus canker when applied in the soil^34–37^. Similarly, we performed experiments to see whether spray applications of SA or MeSA onto leaves could protect plants from Xc infection in greenhouse conditions; however, we observed no significant protection with such treatments, suggesting that SA or MeSA do not penetrate plants when applied as a spray. Thus, future approaches to control citrus canker might consider the induction of basal defense mechanisms to provide a rapid and steady activation of SA synthesis in response to *X. citri* infection. In fact, transgenic citrus plants overexpressing the SA-induced protein kinase (SIPK) homologue CsMAPK1 showed reduced canker pustules, enhanced ROS production and increased levels of *CsPR1* and *CsSAMT*^38^.

In addition to SA and MeSA, RA also inhibited canker symptoms. RA is a caffeic acid ester widely found in plants and thought to accumulate as a defense compound against bacteria and other microbes, in addition to acting as an antioxidant molecule^39,40^. RA has also been shown to mimic a quorum-sensing signal in *Pseudomonas aeruginosa*, however, its role in plant defense against *P. aeruginosa* has not been demonstrated^41^. Because RA did not affect the growth of Xc in culture medium, it is likely that its role as a canker suppressor involves a yet unknown mechanism.

## Methods

### Gene cloning and site direct mutagenesis

The *CsMES1* gene was amplified by reverse transcription (RT)-PCR from total RNA extracted from *C. sinensis* leaves using oligos CATATGGAAGAAGTAGTAGGCATGG and GACCTCTTATGCATACTTAAGAGAAA and cloned into the pGEM-T-Easy vector (Promega). *CsMES1* was subcloned into the *NdeI/EcoRI* site of pET28a vector (Novagen) and moved into BL21 (DE3) cells for protein expression. *CsMES1* was amplified using oligos CATATGGAAGAAGTAGTAGGCATGGAAGAGAAGCATTTTGTTCTAGTTCATGGAGCAAAC and GACCTCTTATGCATACTTAAGAGAAA to produce the CsMES1 V18A mutant. The PCR products were cloned into pGEM-T-Easy vector and subsequently subcloned into the *NdeI/EcoRI* of pET28a for protein expression. Optimized CsMES1 V18S synthetic gene cloned into *NdeI/XhoI* of pET28a was obtained from GenOne Biotechnologies. All constructs were verified by DNA sequencing.

### Protein expression and purification

The *Escherichia coli* BL21 (DE3) cells carrying the recombinant pET28a plasmids were grown at 20 °C for 36–40 h in ZYM-5052 autoinduction media containing kanamycin 50 µg/mL. Cells were pelleted and resuspended in lysis buffer (250 mM NaCl, 10 mM Na_2_HPO_4_, 2.7 mM KCl, 2 mM KH_2_PO_4_, 10% glycerol, 20 mM imidazole, pH 7.5) supplemented with lysozyme (2.5 mg/mL) and sonicated. Cells debris were pelleted, and the soluble proteins were loaded in TALON resin (GE Healthcare Life Science) previously equilibrated with lysis buffer. TALON resin was washed with 20 column volumes of lysis buffer and proteins were eluted with lysis buffer containing 200 mM imidazole. Affinity-purified proteins were further purified by anion exchange chromatography using a HiTrap Q HP 5 ml column (GE Healthcare Life Science) equilibrated and washed with anion exchange buffer (20 mM Tris-HCl, 50 mM NaCl, pH 7.5). Proteins eluted with the anion exchange buffer containing 200 mM NaCl were finally purified on a HiLoad 16/60 Superdex 200 column (GE Healthcare Life Science) equilibrated with SEC buffer (137 mM NaCl, 10 mM Na_2_HPO_4_, 2.7 mM KCl, 2 mM KH_2_PO_4_, pH 7.5). For molecular weight determination, purified CsMES1, as well as the molecular standards (bovine serum albumin, ovalbumin and lysozyme), were loaded on a Superdex 75 10/300 column (GE Healthcare Life Science) equilibrated with SEC buffer. The elution volumes of the molecular standards were used to generate a linear plot of retention coefficient versus the logarithm of the molecular weight. This curve was used to estimate the molecular weight of CsMES1.

### MeSA esterase assay

MeSA esterase activity was assayed according to Park *et al*.^13^ in 0.1 M phosphate buffer (pH 7.0) supplemented with 5 μM β-mercaptoethanol in 96-wells OptiPlates (PerkinElmer) at room temperature. Between 1 and 3 μM of the recombinant CsMES1 proteins in 100 μL of final reaction volume was used. SA and IAA fluorescence were measured using excitation and emission set at 340 and 405 nm, respectively, in an Enspire 2300 multilabel reader (PerkinElmer). For *V*_max_, *K*_m_ and *k*_cat_ estimations, Michaelis-Menten curves were generated using the initial velocity of the reactions obtained with different MeSA concentrations (10 to 500 µM) and fitted using the Hill function ($y={V}_{{\rm{\max }}}\frac{{x}^{n}}{{k}^{n}+{x}^{n}}$). For TriFA IC_50_ determination, the kinetics data from reactions containing 100 μM MeSA and different concentrations of TriFA (10 to 500 µM) were plotted and fitted using the Dose-response function ($y=A1+\frac{A2-A1}{1+{10}^{(LOGxo-x)p}}$). The kinetics data obtained with the increased TriFA concentrations were also plotted using the Lineweaver-Burk format. All calculations and fittings were performed with the OriginLab software.

### Molecular modeling and docking studies

Three-dimensional structural models of CsMES1, CsMES2, CsMES3, and corresponding CsMES1 mutants V18A/V18S were generated by the Swiss-model server^42^ using the SABP2 crystal structure (PDB code 1Y7I) as the search model. For docking studies, charges and polar hydrogen atoms were added to the models using AutoDock Tools (version 1.5.6)^43^ and the prepared models saved as PDBQT files. The TriFA molecule was converted into a PDB file using the Openbabel software^44^, and after Gasteiger charge assignment, the prepared ligand models were saved as PDBQT files. All molecular docking studies were performed using AutoDock4 (version 4.2.6), in which the AutoGrid tool was used for grid map preparation. The Lamarckian Genetic Algorithm (LGA) was chosen to search for best protein-ligand conformers. During the docking process, a maximum of 10 conformers was considered for each ligand. All docking processes were performed with default parameters, except that fluorine atoms were considered as hydrogen bond acceptors. Population size was set to 150, maximum number of evaluations 2,500,000, maximum number of generations 27,000, maximum number of top individual that automatically survived 1, gene mutation rate 0.02 and crossover rate 0.8. All figures with structure representations were produced using the program Pymol v 1.9 (Schrödinger, LLC).

### Isothermal Titration Calorimetry analysis

ITC measurements were performed using the recombinant wild type CsMES1 protein and respective V18A and V18S mutants, purified by affinity and gel filtration chromatography, at concentrations ranging from 20 to 40 μM. The following molecules were tested as CsMES1 ligands: SA (Merck), 2,2,2-Trifluoroacetophenone (Sigma-Aldrich), benzoic acid (Sigma-Aldrich), 3-hydroxybenzoic acid (Sigma-Aldrich) and 4-hydroxybenzoic acid (Sigma-Aldrich) diluted in PBS buffer at final concentrations ranging from 250 μM to 2 mM. The compounds were added to the protein solutions kept in a 2 mL experimental cell using a titration syringe. Ligand titration was performed with 10 μL additions of the ligand with 4-min intervals between additions for temperature stabilization. The experiments were conducted at 20 °C on a VP-ITC MicroCarolimiter (MicroCal) equipped with a VPViewr2000 software (MicroCal) for data acquisition. The recorded data were analyzed by the PEAQ-ITC Analysis Software (MicroCal).

### Bacterial growth and plant inoculations

*Xanthomonas citri* and *X. aurantifolii* pathotype C were grown on LBON medium supplemented with 100 mg/L ampicillin, for 48 h at 28 °C, as described previously^21,22^. Single colonies were suspended in sterile water (OD 600 nm ~0.1) and the suspensions were used to infiltrate sweet orange ‘Natal’ (Washington Navel) plants kept in the green house. Leaves of similar size and age were infiltrated with the bacterial suspensions or water, as control, and after 24 or 48 h, leaf tissues were ground in liquid nitrogen for RNA and metabolite extraction.

For canker symptoms evaluation in the presence of SA, SA analogues and RA, citrus leaves were inoculated with a *X. citri* suspension (OD 600 nm ~0.1) using the pinprick method^45^. After bacterial inoculation, leaves were detached and placed in recipients containing an aqueous solution of the test molecules at 50 µM concentration. The leaves were kept in a plant growth room with a 14 h light photoperiod and temperature oscillating between 25 °C and 28 °C. The solutions containing the test compounds were replaced every two days. Canker pustules were photographed seven days after bacterial inoculation using a Nikon SMZ18 stereomicroscope and the lesion areas, in pixels, were measured using the ImageJ software.

### Gene expression analysis

Total RNA from inoculated and non-inoculated citrus leaves was extracted using Trizol (Invitrogen). The quality and quantity of the RNA samples were verified by agarose gel and UV absorbance. RNA samples treated with DNase I (Promega) were reverse transcribed using the RevertAid Reverse Transcriptase kit (Thermo Fisher Scientific). cDNA samples were diluted and tested for specificity and amplification efficiency relative to the actin gene used as endogenous control^46^. Primer sequences were designed using the NCBI Primer Design Tool (Supplementary Table S1) and PCR reactions were composed of 1x SYBR Green mix, 30 ng cDNA and 7.5 μM forward and reverse primers. Three PCR reactions were performed for each test gene and three biological replicates were analyzed using the universal cycle condition provided by the 7500 System (Applied Biosystems). The results were analyzed by the 7500 System software (Applied Biosystems) using the relative quantification mode and expressed as the mean of nine amplification curves. The treatments were compared using the ANOVA turkey statistical method.

### Extraction and identification of leaf metabolites

For leaf metabolite extraction, approximately 400 μg of leaf tissues inoculated or non-inoculated with *X. citri*, *X. aurantifolii* or water, were macerated in liquid nitrogen and transferred to 2 mL microtubes. The metabolites were extracted by adding 1 mL of 90% methanol to the samples. The powder suspension was homogenized for 1 min and sonicated in a bath for 20 min at room temperature. The suspensions were centrifuged at 5000 × g for 10 min at 4 °C. The supernatants were saved, and the pellets were resuspended in 0.5 mL 100% methanol, homogenized and sonicated as described above. The supernatants were combined and centrifuged at 5000 × g for 10 min at 4 °C. Cyclohexane (0.7 mL) was added, and the mixture was homogenized and centrifuged at 5000 × g for 2 min at 4 °C. The upper phase was discarded, and the cyclohexane extraction repeated twice. The methanolic phase was transferred to a new 1.5 mL tube and dried in a SpeedVac for 1.5 h. Dried samples were stored at −80 °C. For the detection of metabolites, samples were resuspended in 0.3 mL of methanol:water (1:1 v/v), filtered on a 0.22 μm filter and transferred to 0.3 mL vials (Waters). Samples were analyzed on a UPLC Acquity HClass (Waters) hyphenated with a high resolution ESI-QqTOF mass spectrometer detector Impact II (Bruker). Chromatographic separations were performed on a BEH C18 Acquity column (2.1 × 100 mm, 1.7 µm pore size, Waters), at 40 °C and flow rate of 500 µL/min. The solvent system used was water (A), acetonitrile (B) and formic acid 1% (C), following an initial step in 5% B, holding for 1 min, growing to 90% B in 12 min, holding for 1 min and recalibrating for initial step in 2 min. Line C was kept 5% for all 15 min runs. MS data were collected in positive mode with a range of 40 to 1500 Da, using the following parameters: spectra rate of 8 Hz, end plate offset of 500 V, capillary voltage of 4500 V, nebulizer pressure of 4.0 bar, dry gas (N_2_) flow of 10 L/min and dry gas (N_2_) temperature of 200 °C. For fragmentation, the collision cell energy was kept at 5.0 eV whereas the collision energy set between 20 and 70 V, with fragmentation cut-off of 1500 cts and cycle time of 1 s. The acquired MS/MS data were analyzed using the GNPS (Global Natural Products Social) molecular networking approach^47,48^. A network was built based on the GNPS guidelines and reported literature and used to identify the metabolites^47–50^. The fragmentation patterns of SA, 2,3DHBA and RA were confirmed with the respective standards, and the mass peak area used as an estimate of their amount in the samples.

## Supplementary information

Supplementary Information
