# Supplementary material for: Structure-function relationship of a citrus salicylate methylesterase and role of salicylic acid in citrus canker resistance
Source: Sci Rep. 2019 Mar 7;9:3901. doi: 10.1038/s41598-019-40552-3 (PMC6405950; doi:10.1038/s41598-019-40552-3)
Supplement: Supplementary file 1 — Supplementary Information [file 41598_2019_40552_MOESM1_ESM.pdf]

Supplementary Information

Structure-function relationship of a citrus salicylate methylesterase and role of salicylic acid in citrus canker resistance

Caio Cesar de Lima Silva[1], Hugo Massayoshi Shimo[1], Rafael de Felício[1], Gustavo Fernando Mercaldi[1], Silvana Aparecida Rocco[1], Celso Eduardo Benedetti[1]*

[1]Brazilian Biosciences National Laboratory (LNBio), Brazilian Center for Research in Energy and Materials (CNPEM), CEP 13083-100, Campinas, SP, Brazil

Correspondence author information:
E-mail: celso.benedetti@lnbio.cnpem.br
Telephone: (55) 19-35121111
FAX: (55) 19-35121004

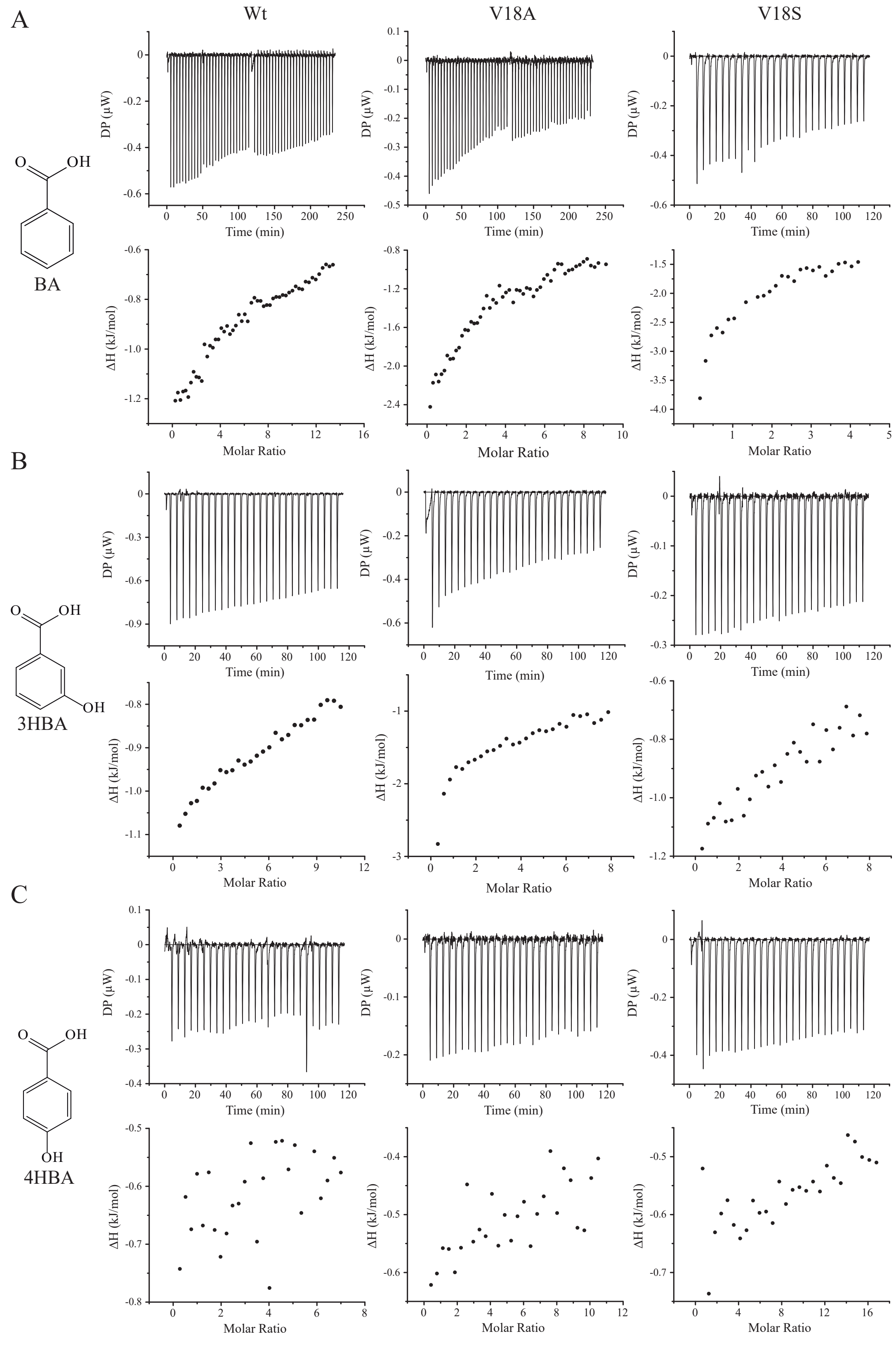

Figure S1. Interaction of wild type and mutant CsMES1 proteins in the presence of SA analogues. ITC measurements of CsMES1 and corresponding V18A and V18S mutant proteins in the presence of BA (A), 3HBA (B) and 4HBA (C), showing that none of the proteins display a measurable binding affinity to any of the SA analogues.

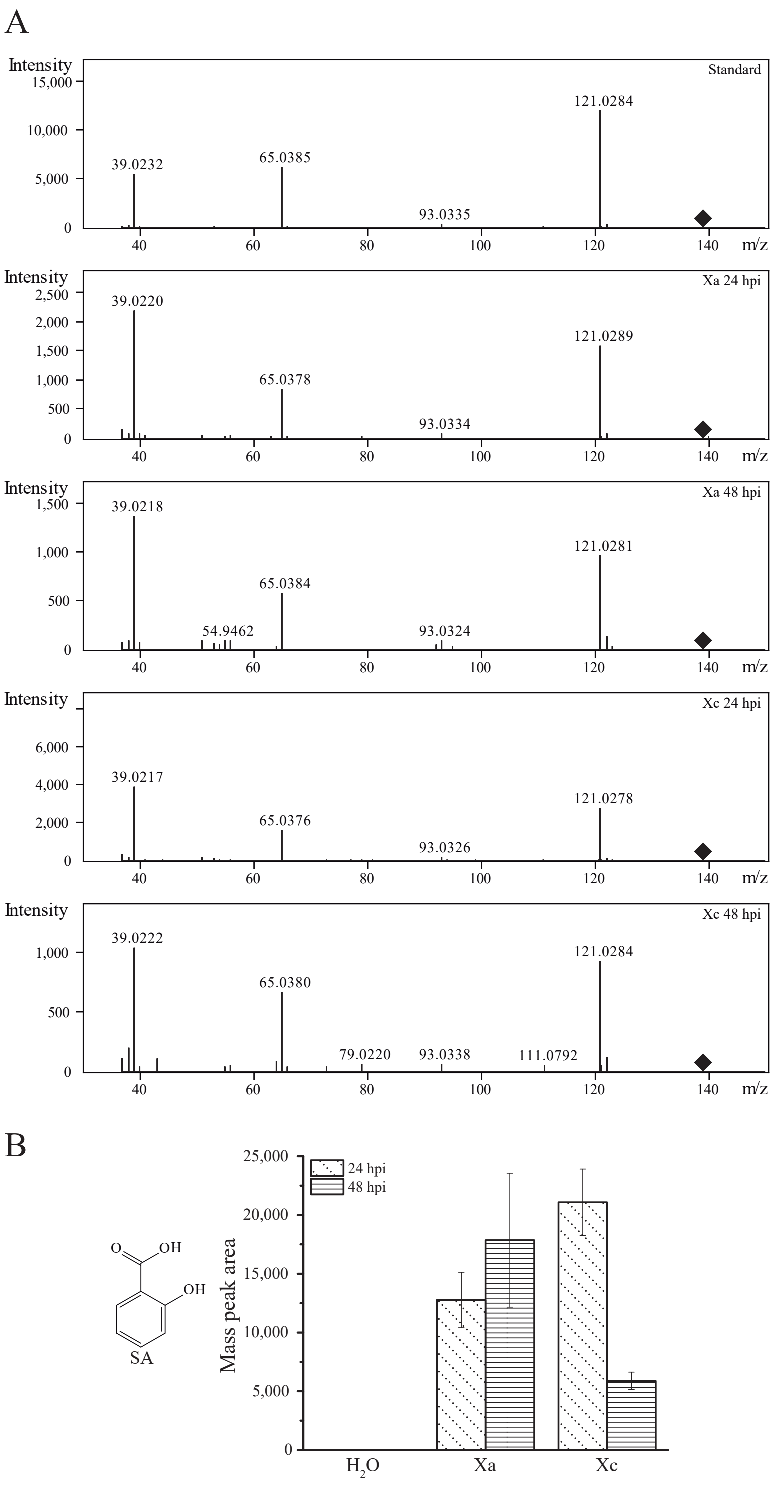

Figure S2. Fragmentation spectra of SA identified in sweet orange leaves infected with Xa or Xc and the amount of SA estimated by high resolution mass peak area. A- Examples of fragmentation spectra of SA from a standard solution (SA from Merck) or from samples of citrus leaves infected with the bacterial pathogens. B- Amount of SA in citrus leaf samples based on peak area, showing that SA is detected at higher levels in Xa-infected leaves at 48 hpi

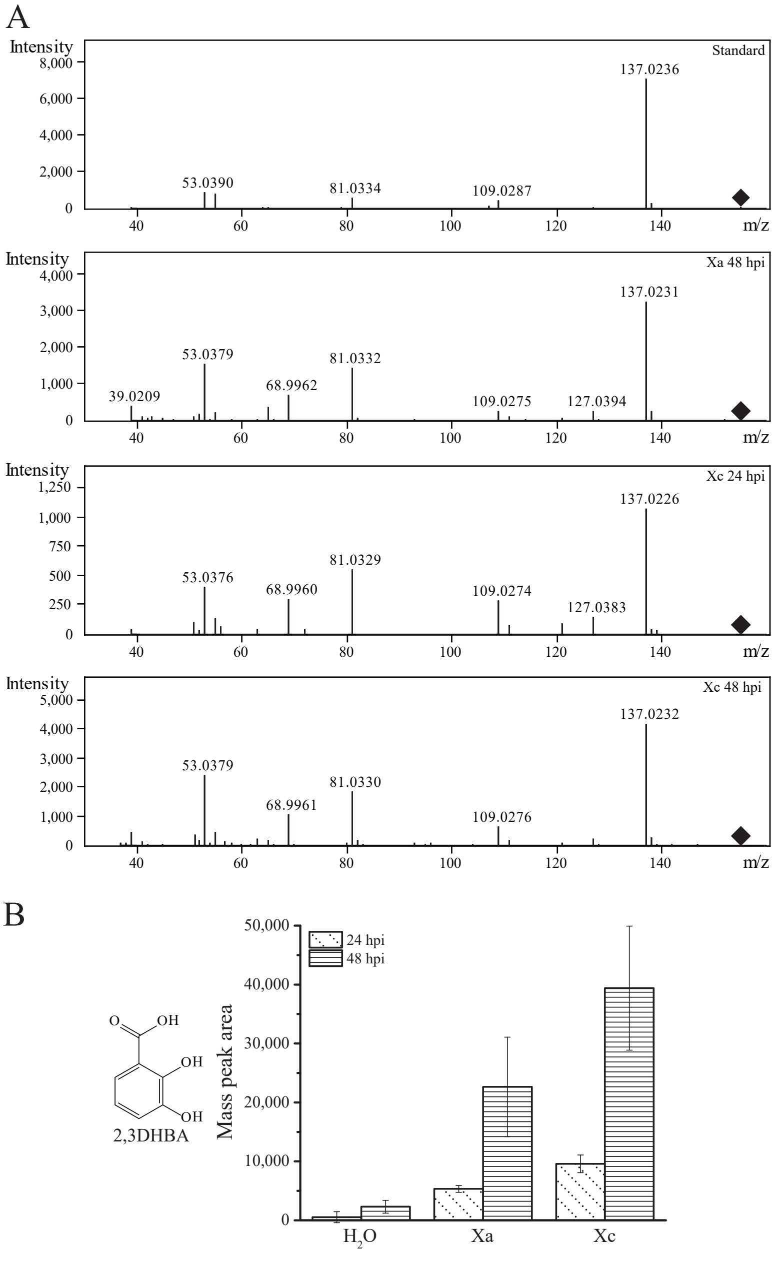

Figure S3. Fragmentation spectra of 2,3DHBA identified in sweet orange leaves infected with Xa or Xc and the amount of 2,3DHBA estimated by high resolution mass peak area. A- Examples of fragmentation spectra of 2,3DHBA from a standard solution (2,3DHBA from Sigma-Aldrich) or from samples of citrus leaves infiltrated with water or the bacterial pathogens. B- Amount of 2,3DHBA in the citrus leaf samples based on peak area, showing that 2,3DHBA is predominantly detected in Xc-infected leaves at 48 hpi.

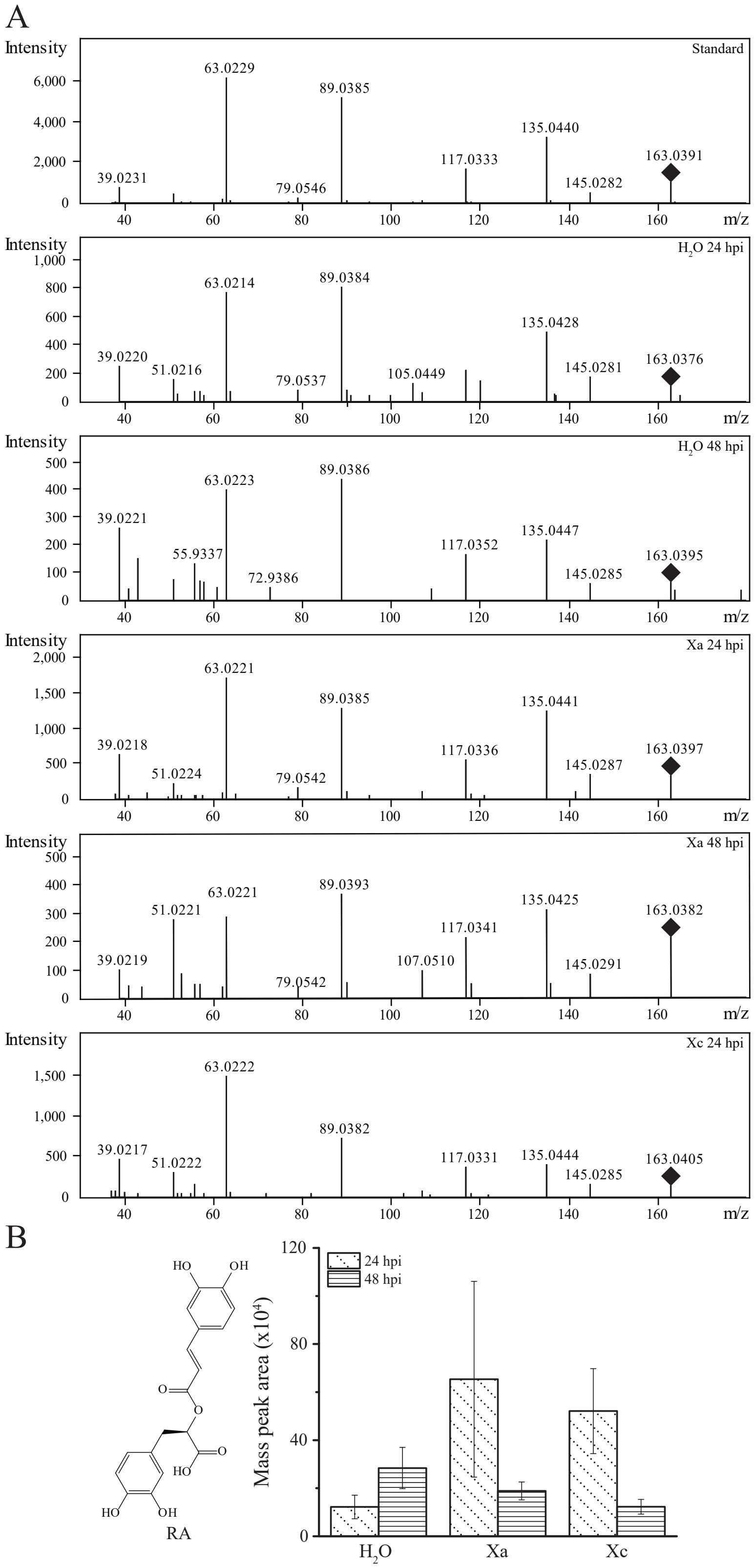

Figure S4. Fragmentation spectra of Rosmarinic acid (RA) identified in sweet orange leaves infected with Xa or Xc and the amount of RA estimated by high resolution mass peak area. A- Examples of fragmentation spectra of RA from a standard solution (RA from Sigma-Aldrich) or from samples of citrus leaves infiltrated with water or the bacterial pathogens. B- Amount of RA in the citrus leaf samples based on peak area, showing that RA accumulates at higher levels in Xa-infected leaves.

Supplementary Table S1. Oligonucleotides used in the RT-qPCR assays.

Oligos	Forward	Reverse	GenBank Number
**CsMES1**	AGAAGTAGTAGGCATGGAAGA	CCAACACCATGCTCATGTTTA	KDO79352
**CsMES2**	AGAAGTAGTAGGCATGAAAGG	CCAACACCATGCTCCATGGTTTG	XP_006466663.1
**CsMES3**	CATTTTGTTCTAGTGCATGGATCA	CGTCACCCGGTGACCGGCTGCCTC	KDO48824
**CsSAMT**	CAACAAGTGTCGGCATTA	ACAAACCAAAGGGCCACA	XM_006466773.3
**CsICS**	GCAGGCACTCCCTCCAAAAA	GGCCATGGACAATGAACACG	XM_006476588.3
**CsC4H**	GCAACGTGGTTTTCGATATT	CAGTGTCATAATCCTACGCA	MM_001288840.1
**CsS3H**	ATACAAGAGTGTGCTCCATC	ACGTTCATCGATCAAATCCT	XM_006490040.3
**CsS5H**	TGGCCTTCAAATCCATCTAC	CTTGTTCACCCAACACTTTC	XM_006478028.3
**CsPR1**	GCAAGGTGTGTGGGCACTATAC	ACCCAATGCGAACCGAATT	XM_006486759.3
**CsNPR1**	GATCCGCTTGTTGCGGTTTT	GGAAAGGCCTCACTTTCCCA	XM_006475416.3
**CsActin**	CCCTTCCTCATGCCATTCTTC	CGGCTGTGGTGGTAAACATGT	XM_006464503.3
